# Plasmids of *Shigella flexneri* serotype 1c strain Y394 provide advantages to bacteria in the host

**DOI:** 10.1186/s12866-019-1455-1

**Published:** 2019-04-29

**Authors:** Pawan Parajuli, Munazza I. Rajput, Naresh K. Verma

**Affiliations:** Division of Biomedical Science and Biochemistry, Research School of Biology, The Australian National University, Canberra, ACT Australia

**Keywords:** *Shigella flexneri*, Plasmids, Virulence, Multidrug resistance, Bacterial pathogen

## Abstract

**Background:**

*Shigella flexneri* has an extremely complex genome with a significant number of virulence traits acquired by mobile genetic elements including bacteriophages and plasmids. *S. flexneri* serotype 1c is an emerging etiological agent of bacillary dysentery in developing countries. In this study, the complete nucleotide sequence of two plasmids of *S. flexneri* serotype 1c strain Y394 was determined and analysed.

**Results:**

The plasmid pINV-Y394 is an invasive or virulence plasmid of size 221,293 bp composed of a large number of insertion sequences (IS), virulence genes, regulatory and maintenance genes. Three hundred and twenty-eight open reading frames (ORFs) were identified in pINV-Y394, of which about a half (159 ORFs) were identified as IS elements. Ninety-seven ORFs were related to characterized genes (majority of which are associated with virulence and their regulons), and 72 ORFs were uncharacterized or hypothetical genes. The second plasmid pNV-Y394 is of size 10,866 bp and encodes genes conferring resistance against multiple antibiotics of clinical importance. The multidrug resistance gene cassette consists of tetracycline resistance gene *tetA*, streptomycin resistance gene *strA-strB* and sulfonamide-resistant dihydropteroate synthase gene *sul2*.

**Conclusions:**

These two plasmids together play a key role in the fitness of Y394 in the host environment. The findings from this study indicate that the pathogenic *S. flexneri* is a highly niche adaptive pathogen which is able to co-evolve with its host and respond to the selection pressure in its environment.

**Electronic supplementary material:**

The online version of this article (10.1186/s12866-019-1455-1) contains supplementary material, which is available to authorized users.

## Background

*Shigella* is a Gram-negative, non-motile, facultative anaerobic human enteric pathogen of the family *Enterobacteriaceae* that is closely related to *Escherichia coli* but has acquired specific traits of pathogenicity, physiology, and antigenic diversity [1]. The key to its virulence is the acquisition of a 210–230 kb virulence plasmid (VP) that enables the bacterium to invade and spread into the intestinal epithelial cells and induce apoptosis in the infected macrophages [2, 3]. There are at least 190 million shigellosis cases and 70,000 deaths annually; principally in developing countries [4]. *S. flexneri* is the primary cause of shigellosis in the developing countries (up to 62% of all *Shigella* spp. infections) [5]. *S. flexneri* has no less than 19 different serotypes based on their antigenic determinants present on the O-antigen of the outer membrane lipopolysaccharide (LPS) [6]. Out of which, *S. flexneri* serotype 1c has emerged as a significant serotype in developing countries over the last decade [7].

Mobile genetic elements including bacteriophages, insertion sequences and plasmids play a very important role in pathogen evolution and genomic plasticity in several bacterial pathogens and *S. flexneri* is not an exception. The VP of *S. flexneri* encodes essential virulence factors regulated by several genes located on the VP as well as on its chromosome [8]. The plasmid comprises a conserved pathogenicity island of 31 kb region which encodes the Ipa-Mxi-Spa type III secretion system (T3SS) [9]. While performing the complete genome sequencing of *S. flexneri* serotype 1c strain Y394 (“Y394” hereafter) [7], we recovered two plasmids which provide pathogenicity advantage to Y394 in the host environment. The first plasmid corresponds to the VP or invasion plasmid of Y394, hence named pINV-Y394. The second plasmid designated as pNV-Y394 possesses a multidrug-resistance cassette. This paper reports the complete nucleotide sequence and gene analysis of the two aforementioned plasmids to further facilitate the understanding of the evolution and pathogenic determinants of Y394, a newly emerged serotype, serotype 1c, of *S. flexneri.*

## Results

### General features of pINV-Y394

**Fig. 1:**
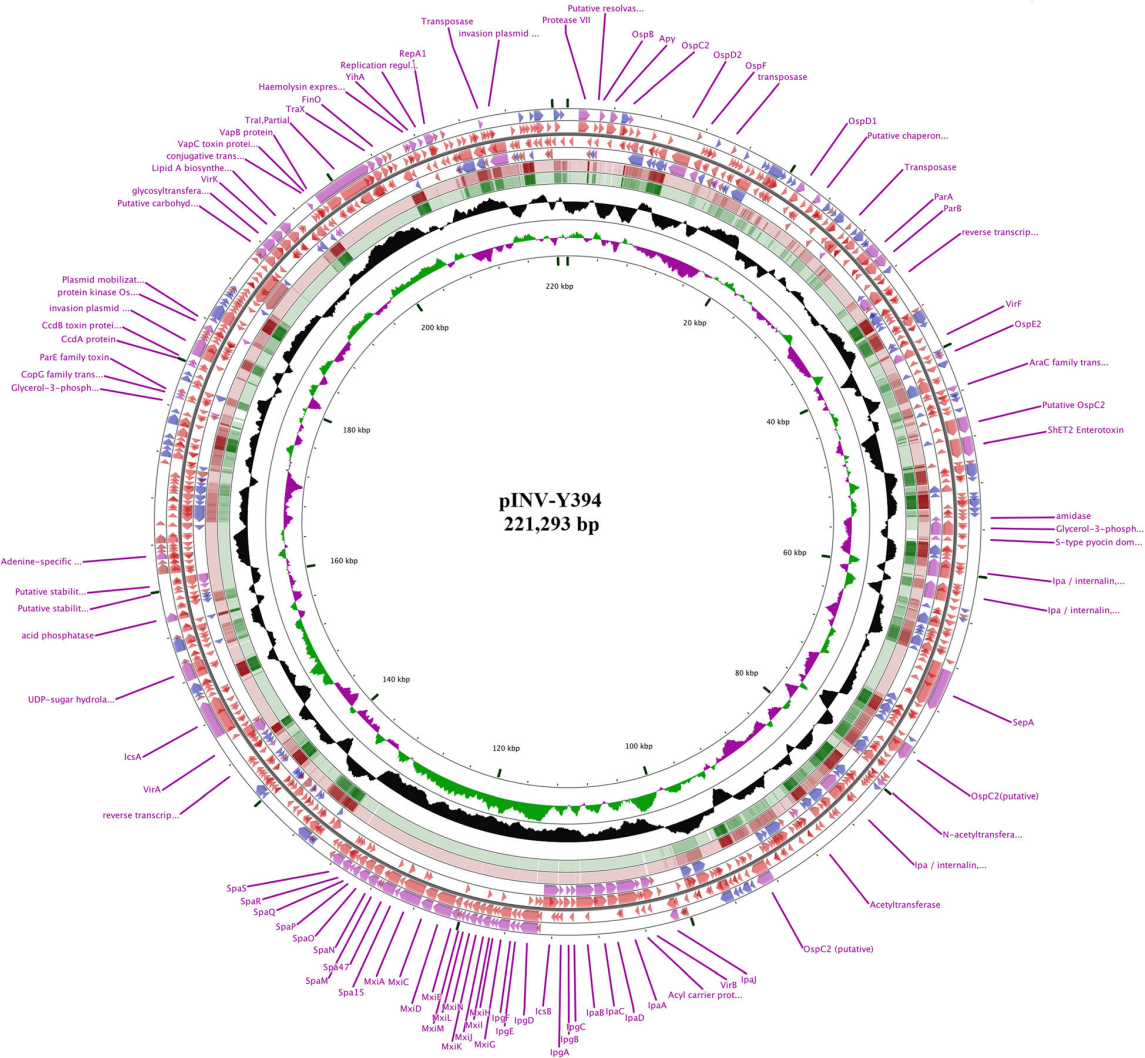
Schematic circular map of the plasmid pINV-Y394. The pair of two outer rings depict ORFs encoded by leading and lagging strands with color codes: virulence associated and known genes (lavender), IS elements (blue) and hypothetical genes (brown). The fifth and sixth rings from outside represent the blast hits of plasmids pCP301 and pWR100. The seventh ring in black shows the deviation from average percentage GC content. The eighth circle in green and purple color denotes the GC skew (G-C/G + C). The innermost ring represents the nucleotide position in the plasmid pINV-Y394

The large VP pINV-Y394 is of size 221,293 bp and comprises of 328 open reading frames (ORFs). The pINV-Y394 is a mosaic of essential virulence genes and their regulons, a significant number of IS elements and several hypothetical genes yet to be characterized (Fig. 1). The GC content of pINV-Y394 was found to be 45.9%, which is lower than that of the Y394 chromosome (50.9%).

The size and the composition, however, are similar to previously published *S. flexneri* serotype 2a VP, pCP301 (GenBank Accession number AL391753); *S. flexneri* serotype 5a VP, pWR100 (GenBank Accession number AF386526) and *S. flexneri* serotype 1a VP, unnamed plasmid1 (referred as “INV-0670” hereafter; GeneBank Accession number CP020087.1) (Table 1).

**Table 1:** General features of *Shigella flexneri* virulence plasmids

Plasmid	Host Strain	Plasmid Size (bp)	GC (%)	ORFs Total	ORFs related to IS	ORFs Uncharacterized	Unique ORFs^a^
pINV-Y394	*Shigella flexneri* 1c strain Y394	221,293	45.9	328	159	72	27
pCP301	*Shigella flexneri* 2a strain 301	221,618	45.8	316	147	78	24
pWR100	*Shigella flexneri* 5a strain M90 T	213,494	45.7	311	138	82	23
unnamed1	*Shigella flexneri* 1a strain 0670	228,834	46.1	301	145	93	35

^a^Unique ORFs refer to ORFs which are present in corresponding plasmid only

**Fig. 2:**
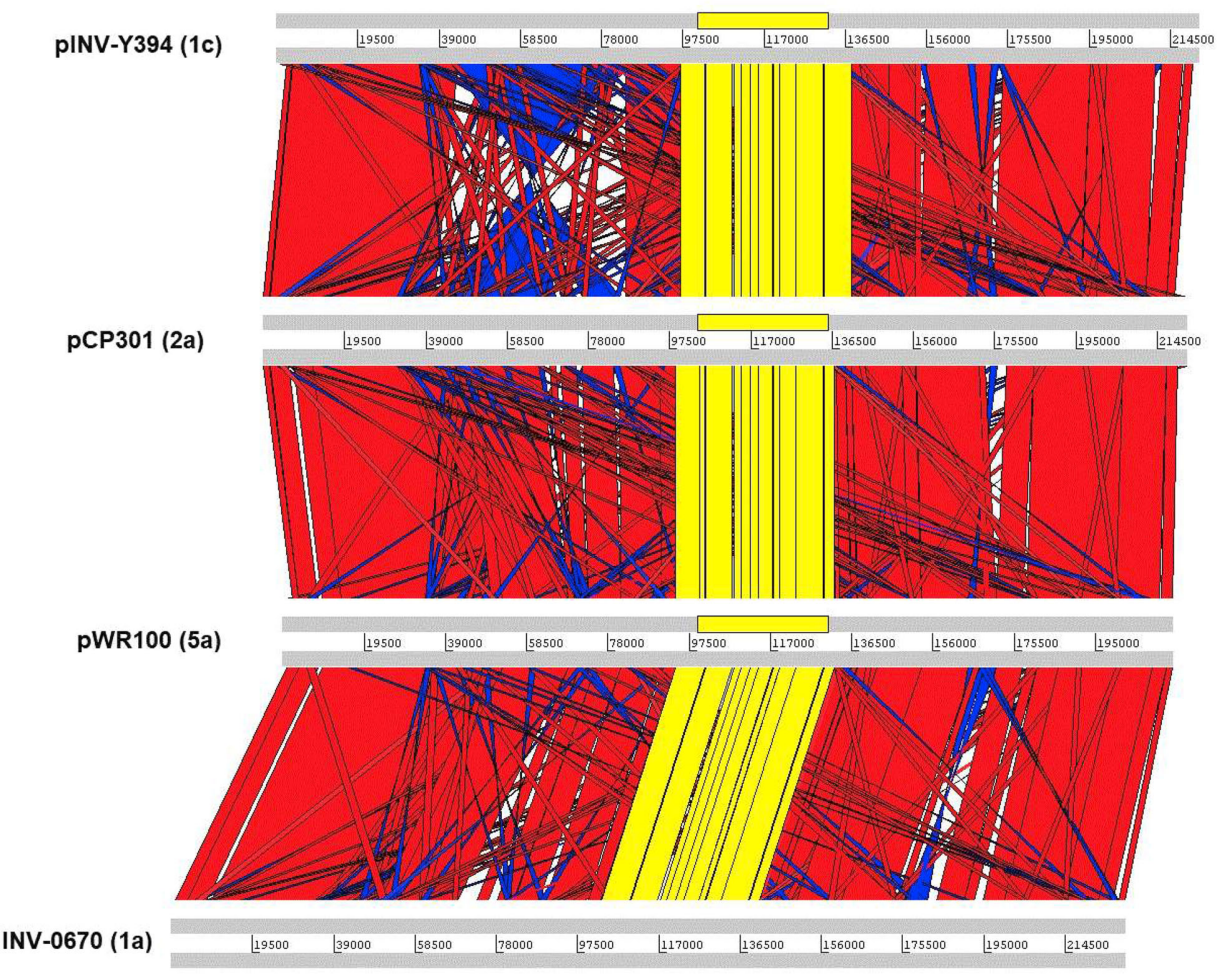
Comparative analysis of the *S. flexneri* virulence plasmids. The compared plasmids are depicted as horizontal grey lines interspersed with regions of collinear (red) and inverted (blue). The regions corresponding to the ipa-mxi-spa T3SS is shaded in yellow. The numbers in the panel indicate the corresponding position in the plasmid sequences

The pINV-Y394 has a similar arrangement of genes as seen in pCP301, pWR100 and INV-0670 with the exception of a large inversion and a few recombination events relative to the pINV-Y394 (Fig. 2). The 31 kb ipa-mxi-spa region is identical in all the compared VPs. We identified 27 unique genes in pINV-Y394 that are not present in three other compared plasmids (with 95% identity cut-off). These genes code for IS elements/transposases (21 ORFs), Osp2 domain protein (1 ORF), conjugation transfer protein (1 ORF) and uncharacterized or hypothetical proteins (4 ORFs).

### IS elements

The sequence analysis of pINV-Y394 identified 159 ORFs related to IS elements, which accounts for 34 % (75,492 bp of 221,293 bp) of the total plasmid sequence. These 159 IS elements belong to 36 different IS families with IS3 being most dominant (76 ORFs) and encode for 52 complete IS elements, 106 partial IS elements and 1 novel putative IS element (Table 2). These IS elements spread all over the genome and usually enclose the virulence-associated genes as well as the uncharacterized ORFs [Fig. 1] with potential virulence function owing to its low GC [10].

**Table 2:** Predicted Insertion Sequence (IS) family in pINV-Y394

IS Family	Total ORFs	Complete IS	Different IS(s)	Total Size
IS1	19	9	4	4806
IS110	3	2	2	2568
IS110 ssgr^a^ IS1111	2	0	2	1140
IS21	4	2	1	4971
IS3 ssgr IS2	11	3	4	4953
IS3 ssgr IS3	32	11	4	11,718
IS3 ssgr IS51	28	8	1	11,124
IS3 ssgr IS150	5	1	3	1731
IS4 ssgr IS4	3	3	1	3960
IS4 ssgr IS10	2	1	1	1527
IS4 ssgr IS50	1	0	1	1116
IS5 ssgr IS427	7	3	1	2547
IS66	18	8	4	12,699
IS91	20	0	5	8376
IS630	2	1	1	1617
ISL3	2	0	1	639
Total	159	52	36	75,492

^a^ssgr -subgroups

### Virulence associated genes

The 31 kb region of ipa-mxi-spa genes lies at position 100,933 bp-132,292 bp of pINV-Y394. This region comprises of 39 coding sequences clustered together and is transcribed in two directions. The 12 genes from *ipaJ to icsB* are transcribed in one direction whereas 27 genes from *ipgD* to *spaS/spa40* are in the opposite direction. The GC content of this region is 34.22% [range: 27% (ORF180; hypothetical gene)- 39% (*Spa47/IpaC*)] which is lower than the overall GC content of the plasmid. The 39 ORFs include five *ipa*, six *ipg*, twelve *mxi*, nine *spa*, *virB, ACP* (Acyl Carrier Protein), *icsB* and four uncharacterized genes. The ipa-mxi-spa region is flanked by truncated transposase on both the sides suggesting its origin from a single entity. The downstream to *spaS* lies a hypothetical gene (32% GC) containing conserved protein domain family *YmgB.* YmgB/AriR protein has been shown to be critical for biofilm formation and acid resistance in *E. coli* [11].

Besides the ipa-mxi-spa region, pINV-Y394 also possesses several other genes which might have a potential role in virulence. There are eleven putative *osp* genes (*ospB, ospC1, ospC2, ospC3, ospD1, ospD2, ospD3, ospE1, ospE2, ospF, ospG*) distributed across the plasmid genome. Several of these genes including *ospB, ospC1, ospE2,* and *ospF* have been shown to be regulated by the protein MxiE and is believed to have a role in post-invasion steps of infection [12].

Besides the invasion plasmid antigen (*ipa)* genes in the ipa-mxi-spa region, there are five more alleles of *ipaH* genes named as *ipaH1.4, ipaH2.*5, *ipaH4.*5, *ipaH7.8,* and *ipaH9.8.* These genes possess a novel E3 ligase (NEL) domain at the C-terminus and a series of leucine-rich repeats at the N-terminus which are found in many bacterial virulence factors. The leucine-rich repeats sequester a cysteine residue contained in the NEL domain until invasion has occurred allowing the release of NEL domain for site-specific function [13, 14].

The key regulatory gene for virulence function, *virF,* is located approximately 62 kb upstream of *virB* of the ipa-mxi-spa locus. The activation of the plasmid-encoded regulatory genes requires environmental stimuli such as temperature, osmolarity, and changes in DNA supercoiling [8, 15, 16]. The VirF protein binds to the promotor of *virB* which acts as a transcriptional activator of the genes of the T3SS [17]. Approximately 12-kb downstream of *spaS* lies the *virA* and *icsA/virG* with a distance of 0.5-kb between them. The product of these genes contributes to the intracellular spread and protection against autophagic degradation [18, 19]. The other *vir-*gene, *virK,* which is located 43-kb downstream to *virG* is required for post-transcriptional control of the *virG* gene [20].

### Genes associated with plasmid maintenance and transfer functions

The sequence analysis also identified at least five sets of genes related to toxin-antitoxin (TA) systems. This includes *parAB, ccdAB, stbAB, vapBC and parE/copG.* These genes are essential for maintaining the plasmids in bacteria through post-segregational killing mechanisms which eliminate bacterial cells lacking a plasmid after cell division. Besides, these genes are also involved in multiple cellular functions associated with survival under stress conditions [21].

pINV-Y394 consisted of several truncated conjugative transfer genes suggesting that it had conjugative transfer system but had lost its essential components over time. The plasmid possessed only 6 % (45 AA) of the fully functional TraD (723 AA) which is an essential component of DNA transfer in bacterial conjugation system [22]. The pINV-Y394 lacks the essential region of TraD, the 38 amino acids at the very C-terminal end of the protein. The *traI* gene in pINV-Y394 has a deletion leading to a frameshift mutation. The two other genes present in pINV-Y394 related to conjugation are *traX* and *finO* which code for pilus acetylation protein and fertility inhibition protein. The FinO is one of the components of FinOP fertility inhibition complex. This protein inhibits the expression of *traJ* gene which in turn regulates the expression of some 20 other transfer genes. However, the remainder of the transfer genes was absent in pINV-Y394. The repeated conjugation experiments with pINV-Y394 were unsuccessful and are consistent with the previous observation [23].

### Plasmid pNV-Y394

**Fig. 3:**
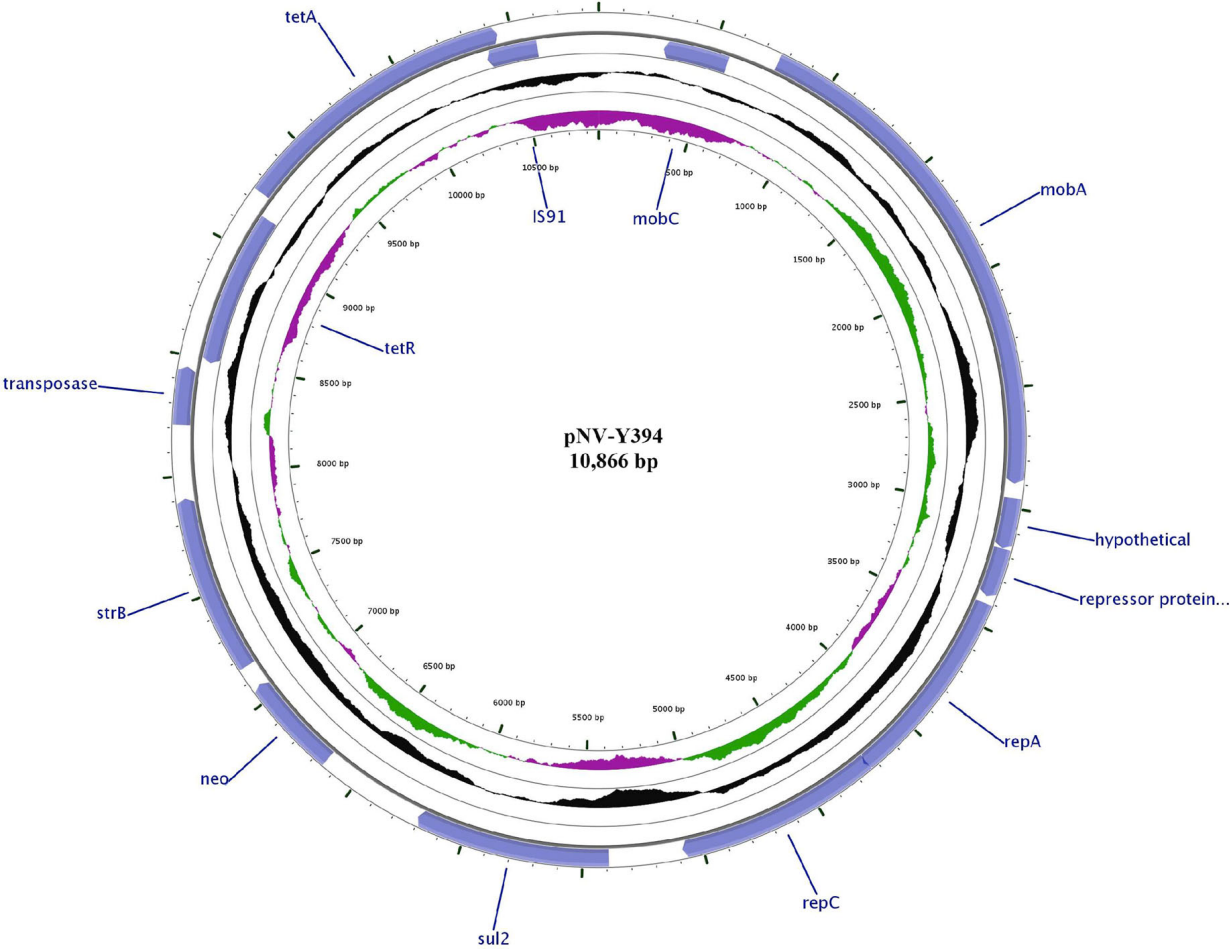
Schematic circular map of the plasmid pNV-Y394. The two outer rings depict ORFs in pNV-Y394 (blue). The third ring in black shows the deviation from average percentage GC content. The fourth circle in green and purple color denotes the GC skew (G-C/G + C). The innermost ring represents the nucleotide position in the plasmid

**Fig. 4:**
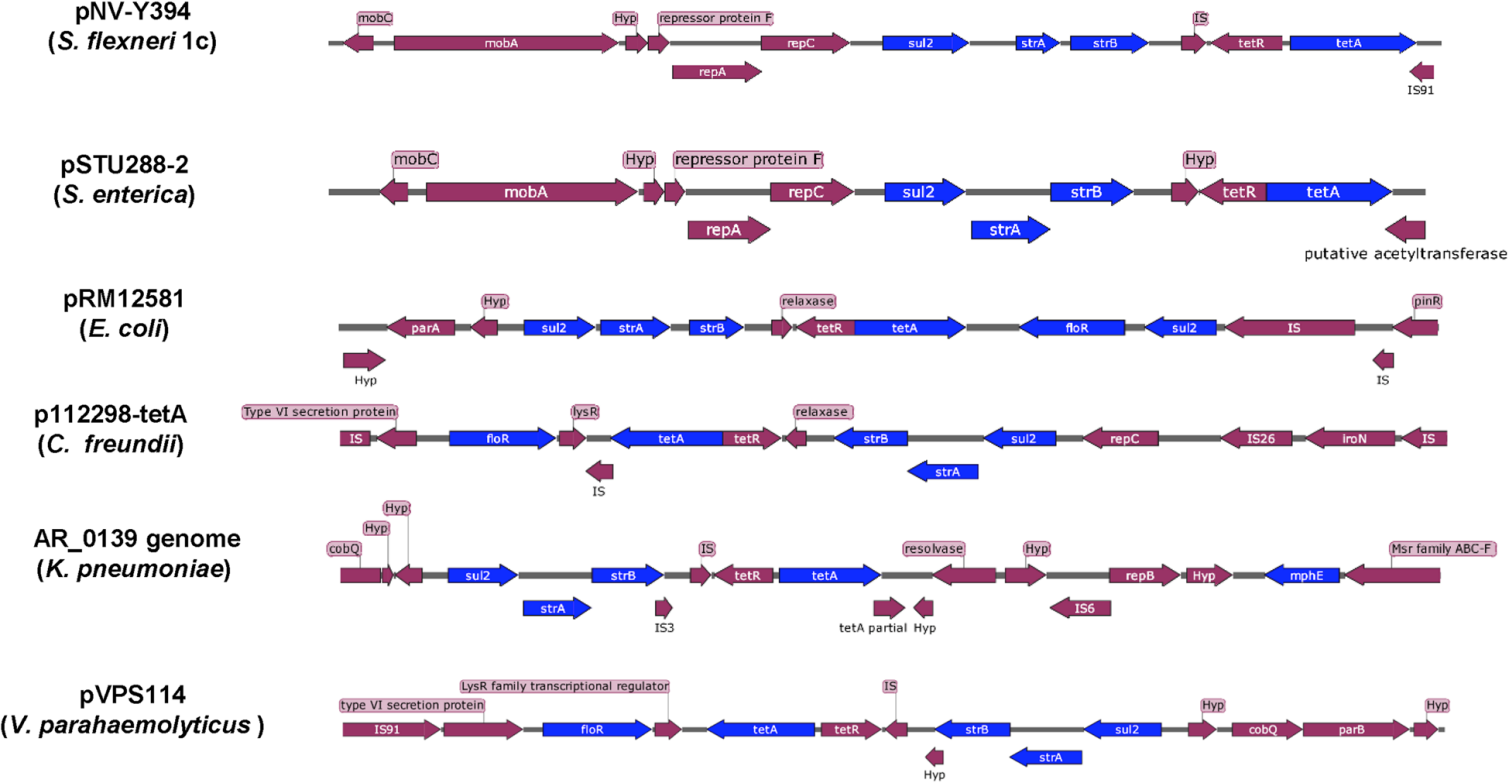
Genomic organization of the plasmid pNV-Y394 and its homologues. The arrows indicate the direction of genes coded by forward and reverse strand on each of the compared plasmids/genome. The genes conferring drug resistance are higlighted in blue

The plasmid pNV-Y394 is 10,866 bp in size and has GC content of 61.54% which is well above the 51% GC content of the host bacteria Y394 (Fig. 3). The plasmid encodes multiple antibiotic resistance genes of clinical importance (Table 3). The plasmid is novel in *S. flexneri* but has partial homology of multidrug-resistance genes cassette (from nucleotides 5091 to 10,715) with other bacterial genomes and plasmids. The BLASTN result showed 100% query cover and 99% nucleotide identity with genomes and plasmids of several other clinical isolates including *Salmonella enterica* (GenBank accession numbers CP022498.1 and CP004059.1), *Citrobacter freundii* (GenBank accession number KY986974.1), *Escherichia coli* (GenBank accession number CP007137.1), *Klebsiella pneumoniae* (GenBank accession number CP021960.1) and *Vibrio parahaemolyticus* (GenBank accession number KY014465.1). The arrangement of genes in 12 kb region of the aforementioned genomes/plasmids along with 5 kb region of homology is shown in Fig. 4.

**Table 3:** Features of pNV-Y394

Feature/gene	Start	Stop	Length (bp)	Function
*mobC*	569	285	285	Mobilization protein C
*mobA*	768	2897	2130	Conjugal transfer protein TraA
Hypothetical protein	2958	3170	213	Hypothetical protein (Multispecies of Bacteria)
Repressor protein F	3172	3378	207	Major facilitator superfamily MFS_1
*repA*	3408	4247	840	IS, phage, Tn; Transposon-related functions
*repC*	4234	5085	852	Plasmid replication protein C
*sul2*	5393	6208	816	Dihydropteroate synthase (EC 2.5.1.15)
*strA/neo*	6653	7072	420	Aminoglycoside 3′-phosphotransferase (EC 2.7.1.95) Streptomycin 3′-kinase StrA (EC 2.7.1.87)
*strB*	7164	7907	744	Aminoglycoside 3′-phosphotransferase 2 (EC 2.7.1.95) Streptomycin 3′-kinase StrB (EC 2.7.1.87)
Putative transposase	8213	8455	243	Putative transposase/relaxase /helicase
*tetR*	9164	8487	678	Transcriptional regulator, TetR family
*tetA*	9243	10,442	1200	Tetracycline efflux protein TetA
IS91	10,373	10,597	225	IS91 family transposase

The multidrug cassette consists of genes *sul2, strA-strB* and *tetA* which confers resistance against sulfonamide, streptomycin, and tetracycline, respectively. The phenotype was consistent with the genotype of pNV-Y394. The phenotypic expression of these antibiotic resistance genes was also confirmed with *E. coli* JM109 cells transformed with pNV-Y394.

Although pNV-Y394 harbored genes *mobA* and *mobC* associated with plasmid transfer and *repA* and *repC* associated with plasmid replication, we were unable to show the plasmid transfer by conjugation experiments, suggesting these genes are not sufficient for conjugative transfer.

## Discussion

The large VP pINV-Y394 in *S. flexneri* serotype 1c strain Y394 encodes a 30-kb ipa-mxi-spa locus consisting of type III secretion system (T3SS) and an array of virulence factors which enable the bacteria to initiate invasion and subsequent interaction with the host epithelial cells and adapt intracellular lifestyle [2]. While the *ipa*, *mxi* and *spa* genes are mostly involved in early stage of infection, the *osp* and *ipaH* genes are involved in post-invasion events [12]. The *ipaH* family genes are present on both the VP and the *Shigella* chromosome [24] and are capable of subverting the host’s ubiquitination pathway [13, 14].

The low GC percentage of pINV-Y394 suggests that the pathogen has acquired the plasmid later as a response to adaptive evolution. There are a large number of IS elements in pINV-Y394 which accounts over one-third of the total plasmid genome. Most of the genes associated with virulence have lower GC content and are flanked by the IS elements suggesting that these genes have been brought as the result of numerous horizontal gene transfer via IS-mediated recombination events. The comparison of VP sequences from three other serotypes of *S. flexneri* showed a high level of similarity with some recombination and a large inversion [23, 25]. The ipa-mxi-spa region was highly conserved in all the compared plasmids and is flanked by the IS elements at both the ends suggesting that these genes have a common origin and was acquired by IS-mediated recombination. Further, the few differences in gene content of the compared plasmids were mostly in the sequence of IS elements; suggesting all the compared plasmids had a common backbone. Out of 328 ORFs, about 26 % of them are uncharacterized in pINV-Y394. Many of which have low GC content and flanked by IS elements, suggesting that these genes are foreign and might have brought to the plasmid by several IS-mediated recombination events. These uncharacterized genes warrant further studies on their function(s).

The lack of conjugative transfer of pINV-Y394 is consistent with previous studies [23, 25] and can be explained by the loss of intact *tra* locus which is essential for the exchange of plasmids between two bacteria by conjugation. The TraD protein which acts as a coupling protein and connects the DNA-processing machinery to the mating pair-forming transfer apparatus is truncated in pINV-Y394 with the loss of 38 amino acid C-terminal region essential for TraM interaction [22]. The genes *traD, traM,* and *traY* are involved in the *oriT* nicking, strand displacement, and DNA transfer during conjugation [26].

The other important gene of the *tra* locus, *traI,* has a frameshift mutation in its coding sequence. The protein TraI is a bifunctional protein which acts as a sequence-specific DNA trans-esterase, providing the site- and strand-specific nick required to initiate DNA transfer and a processive 5′ to 3′ helicase reaction that provides the motive force for strand transfer [27]. The loss of transfer function in VP has stabilized the virulence genes alongside allowing parallel evolution of the VPs with the chromosomes [28].

Although, no other *S. flexneri* strain has been previously reported with multidrug resistance region as found in pNV-Y394, the 5-kb antibiotic resistance cassette of plasmid pNV-Y394 which provides resistance to the bacteria against sulfonamide, streptomycin and tetracycline antibiotics of clinical importance, has been isolated from several bacterial pathogens including *S. sonnei* [29], *Salmonella enterica* [30] and *Klebsiella pneumoniae* [31]. The antibiotic resistance cassette in pNV-Y394 is flanked by IS91 family transposases, which are linked to active rolling-circle transposition of several virulence and toxin genes and play an important role in pathogen evolution [32, 33]. Although, pNV-Y394 lacks complete conjugative transfer elements and is non-conjugative, the antibiotic gene cassette might have brought by IS91-mediated gene dissemination into Y394. The horizontal acquisition of multiple drug resistance genes has been suggested to play an important role in driving the global spread of a pathogen as a new epidemic clone [34, 35]. The increase in multiple drug resistance strains of *S. flexneri,* endemic in developing countries, is of major concern due to limited resource settings for antibiotic susceptibility testing and limited therapeutic options. The antibiotic resistance surveillance studies across multiple *Shigella* endemic regions including China, India, Bangladesh, Pakistan, Nepal, Gabon, Kenya, Senegal, Iran and Egypt showed high percentage of *S. flexneri* isolates being resistant to tetracycline, trimethoprim-sulfamethoxazole, ampicillin and streptomycin [36–48]. This warrants the choice of antibiotic therapy be based on recent antibiotics susceptibility data of *Shigella* isolated from nearby geographical regions.

## Conclusions

The comparison of pINV-Y394 with pCP301, pWR100 and INV-0670 showed that all four plasmids were highly conserved despite they were derived from different *S. flexneri* serotypes. This suggests that VP is a prerequisite for *S. flexneri* virulence and might have been acquired from a common ancestor. Further functional characterization of the conserved hypothetical genes would increase our understanding of virulence gene regulatory networks. The VP pINV-Y394 has the essential genetic element for its virulence and pNV-Y394 provides resistance to Y394 against an array of antibiotics of clinical importance. Hence, the two plasmids isolated from Y394 have an important role in the fitness of Y394 in the host environment. Furthermore, proper surveillance of antimicrobial resistance of this globally important pathogen is warranted, as this information is crucial for effective outbreak management and control of the disease.

## Methods

### Bacterial strains and plasmids

The two plasmids pINV-Y394 and pNV-Y394 presented in this study were derived from *S. flexneri* serotype 1c strain Y394 which was kindly provided to us by Nils I. A. Carlin [49]. The Y394 was grown aerobically (180 rpm) at 37 °C in Luria Bertani broth (LB). The total bacterial DNA was prepared using the Genomic Tip 100/G (Qiagen) according to the manufacturer’s instructions. The LB was supplemented with antibiotics (Sigma-Aldrich, Germany) where needed. The antibiotic susceptibility pattern of Y394 was determined as described previously [7].

### Plasmid DNA sequencing and assembly

The complete genome of *S. flexneri* serotype 1c strain Y394 was sequenced using the hybrid method of single-molecule real-time (SMRT) sequencing technology and short-read MiSeq (Illumina) sequencing technology as described previously [7]. Briefly, The SMRTbell Template Prep Kit 1.0 (PacBio) was used for SMRT sequencing library preparation of about 20 kb insert size. The sequencing was performed using PacBio RSII sequencing system. The Nextera XT DNA library preparation kit (Illumina) was used for MiSeq v3 300bp paired-end sequencing. A de novo assembly of these reads was performed with HGAP.3 (Pacific Biosciences) on the SMRT Analysis Pipeline version 2.3.0 [50]. Following de novo assembly of the SMRT reads, a single contig of 4,584,634 bp (203X coverage) representing the Y394 chromosome was obtained (GenBank Accession number CP020753). The sequence reads that did not map to the chromosome were then independently assembled into two contigs of size 221,307 bp (250X coverage) representing pINV-Y394 and 10,873 bp (30X coverage) representing pNV-Y394. The circularization of the plasmid was obtained using a package Circlator [51]. To improve the quality of the SMRT sequences, the miseq reads were quality trimmed using Trimmomatic v0.36 [52] and used for error correction by using BWA-MEM and Pilon [53, 54]. After circularization and Pilon improvement the final size of pINV-Y394 was 221,293 bp and pNV-Y394 was 10,866 bp.

### DNA sequence annotation and analysis

The automated annotation of plasmid sequences was performed using Rapid Annotation using Subsystem Technology (RAST) version 2.0 [55] followed by manual curation using BLASTP searches [56]. The IS elements were identified using ISsaga [57]. The antibiotic profiles of the plasmids were examined with ResFinder [58]. The genes that were common and unique in each of the compared plasmids were analysed using Roary [59].

The image files showing the synteny map of the plasmids were obtained using ACT (Artemis Comparison Tool) [60], CG View Server [61] and Snap Gene Viewer (Version 3.3.1).

### Plasmid characterization and tagging

The plasmid pINV-Y394 was first tagged with *kan* gene for antibiotic selection using λ-red recombinase system as described previously [62]. Briefly, the Y394 strain was first transformed with pKD46 plasmid encoding λ-red recombinase system. Fifty-base pair overhang primers (Sigma, Australia) were designed corresponding to the putative reverse transcriptase gene of pINV-Y394 and used for amplification of *kan* gene using pKD4 miniprep DNA as a template (Additional file 1: Table S1). The PCR amplicons were then DpnI-treated; column purified and transformed with Y394 + pKD46 electrocompetent cells. The transformants were selected using LB agar plates containing kanamycin (50 μg/ml) and confirmed by Sanger sequencing.

To examine the phenotypic expression of antibiotics resistant genes in pNV-Y394, miniprep DNA was extracted using Wizard® Plus SV Minipreps DNA purification system (Promega, USA) and the plasmid preparations were used to transform electrocompetent *Escherichia coli* JM109 cells and the resultant clones were recovered using LB-agar plates containing either tetracycline (10 μg/ml) and/or streptomycin (10 μg/ml).

### Conjugation

To determine the ability of conjugative transfer of the two plasmids, mating experiments were performed. The mating experiment was carried out by mixing the exponential growth phase cultures of donor *S. flexneri* serotype 1c strain Y394 and recipient *E. coli* JM109 transformed with pBCSK (+) containing chloramphenicol-resistance gene (*Cm*^*r*^) to aid in antibiotic selection. The mating mixture was incubated in a 37 °C water bath for 1 h and resuspended in PBS. The serial dilutions were then plated on LB plate containing either kanamycin (50 μg/ml) and chloramphenicol (25 μg/ml) or tetracycline (10 μg/ml) and chloramphenicol (25 μg/ml) to discriminate the conjugative transfer of plasmids pINV-Y394 and pNV-Y394, respectively.

## Additional file

Additional file 1: **Table S1.** Primers used in this study. (PDF 89 kb)

## Abbreviations

GC: Guanine and cytosine
IS: Insertion sequences
T3SS: Type III secretion system
VP: Virulence plasmid
