# Supplementary material for: Plasmids of Shigella flexneri serotype 1c strain Y394 provide advantages to bacteria in the host
Source: BMC Microbiol. 2019 Apr 29;19:86. doi: 10.1186/s12866-019-1455-1 (PMC6489325; doi:10.1186/s12866-019-1455-1)
Supplement: Supplementary file 1 — Table S1. Primers used in this study. (PDF 89 kb) [file 12866_2019_1455_MOESM1_ESM.pdf]

Table S1. Primers used in this study.

Primers	Sequence (5’-3’)	Purpose
**VP_Kan**	F:atttgatgcacgtagtaaataccagtgaacaaatagcttcggatactcct**cttgagcgattgtgtaggct**<br>R:gatgcgacgggcagactttgaaaatgttcgaccagcaacgtgatggcaatt**tgaatatcctccttagttcc**	50 bp overhang primers for tagging virulence plasmid with *kan*
**KO_test**	F: cgccttcttgacgagttctt<br>R: ctaaggaatgaggtggtgag	Verification of transformation
